# Characterization of the complete mitochondrial genome of the *Pohlia nutans* M211 from Antarctica

**DOI:** 10.1080/23802359.2020.1780980

**Published:** 2020-06-17

**Authors:** Junhan Cao, Xihong Yang, Yanfeng Wang, Yingying He, Changfeng Qu, Jinlai Miao

**Affiliations:** a College of Marine Science and Biological Engineering, Qingdao University of Science and Technology, Qingdao, China; b Key Laboratory of Marine Eco-Environmental Science and Technology, First Institute of Oceanography, Ministry of Natural Resource, Qingdao, China; c Laboratory for Marine Drugs and Bioproducts, Qingdao Pilot National Laboratory for Marine Science and Technology, Qingdao, China

**Keywords:** *Pohlia nutans* M211, mitochondrial genome, phylogenetic analysis

## Abstract

The complete mitochondrial genome of *Pohlia nutans* M211, sequenced using Illumina NovaSeq PE150, was 99864 bp in length. It encoded 65 genes, comprising 38 protein-coding genes, 24 tRNA genes and 3 ribosomal RNA genes. For these 38 PCGs, the most common start codon was ATG and the most common termination codon was TAA. The total GC content was 36.91% while the composition of A + T was 60.39%. Phylogenomic analysis indicated that *P. nutans* M211 was closely related to *Mielichhoferia elongate*.

Antarctica is the coldest, driest and most hostile continent in the world (Pearce 2008; Jin et al. 2020). Moss is one of the major macrophytes of Antarctic terrestrial ecosystems (Casanovas et al. 2013). Facing the extreme environments conditions, this plant has evolved a high degree of adaptability at the molecular and cellular level which exhibited the genetic diversity (Alberdi et al. 2002). However, the mechanism of it remains unclear. Therefore, we sequenced the complete mitochondrial genome of *Pohlia nutans* M211 to provide more data information for further research.

*P. nutans* M211 (Accession no. FIO2008625801), collected from the Great Wall station in Antarctica (62°12′59 S, 58°57′52 W), was stored in the Key Laboratory of Marine Eco-Environmental Science and Technology, the First Institute of Oceanography, Ministry of Natural Resource, China. The whole genome was extracted by the SDS method (Lim et al. 2016) and sequenced by the Illumina NovaSeq PE150 at the Beijing Novogene Bioinformatics Technology Co., Ltd. In addition, it was assembled by SOAP DeNovo software and annotated by the GeneMarkS (Besemer et al. 2001).

The complete mitochondrial genome of *P. nutans* M211 (Genbank accession number: MN956803) was 99864 bp in length and encoded 65 genes, including 38 protein-coding genes (PCGs), 24 tRNAs, and 3 rRNAs. For these 38 PCGs, the most common start codon was ATG (ccmFN, rps3, rpL2, atp1, ccmFC, ccmC and rps1) while the most common termination codon was TAA (ccmFN, rps3, nad2, nad4, and ccmC). The genome composition was 30.17% for A, 30.22% for T, 19.17% for C and 20.43% for G, with a distinct (A + T) % > (G + C) %. Moreover, the total GC content was 36.91%.

To verify the phylogenetic location of *P. nutans* M211, complete mitochondrial genome sequences from 13 species of Bryidae and 1 species of Bryophytina were selected to constructed phylogenetic trees by MEGA7.0 under Maximum Likelihood method with 1000 bootstrap calculations (Kumar et al. 2016). It was indicated that the phylogenetic relationship of *P. nutans* M211 was very close to *Mielichhoferia elongate* (Figure 1). This study laid a foundation for revealing the adaptation mechanism of Antarctic moss to extreme environments and promoting the development of special genetic resources in polar regions.

**Figure 1.**
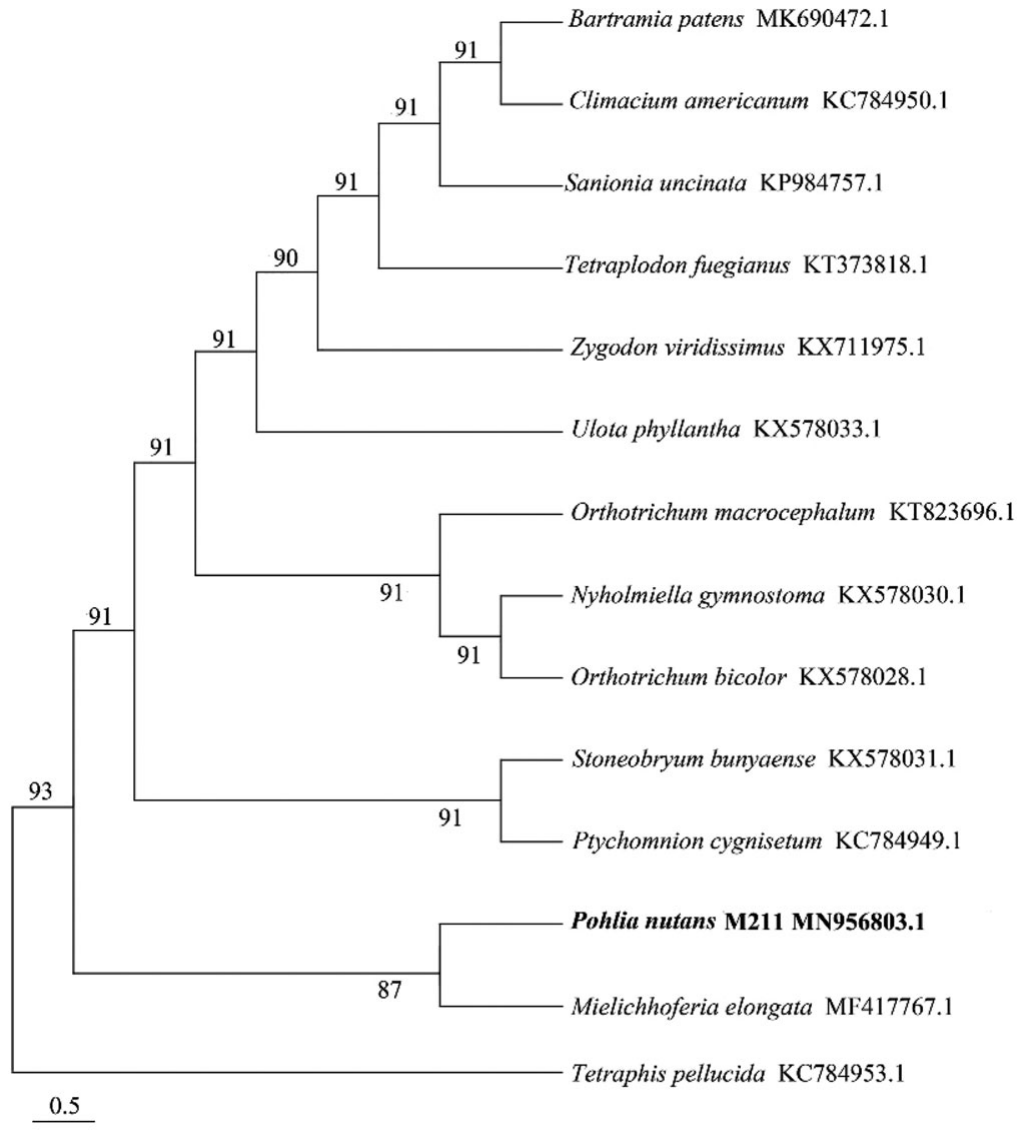
Phylogenetic tree based on 14 complete mitochondrial genomes.

## Data Availability

The data that support the findings of this study are openly available in the NCBI at https://www.ncbi.nlm.nih.gov/, reference number is MN956803.
